# Optic Atrophy and Inner Retinal Thinning in *CACNA1F*-Related Congenital Stationary Night Blindness

**DOI:** 10.3390/genes12030330

**Published:** 2021-02-25

**Authors:** Kate E Leahy, Tom Wright, Monika K Grudzinska Pechhacker, Isabelle Audo, Anupreet Tumber, Erika Tavares, Heather MacDonald, Jeff Locke, Cynthia VandenHoven, Christina Zeitz, Elise Heon, J Raymond Buncic, Ajoy Vincent

**Affiliations:** 1 Department of Ophthalmology and Vision Sciences, The Hospital for Sick Children, Toronto, ON M5G 1X8, Canada; kate.leahy@uon.edu.au (K.E.L.); m.g.pechhacker@gmail.com (M.K.G.P.); anupreet.tumber@sickkids.ca (A.T.); heather.macdonald@sickkids.ca (H.M.); jeff.locke@sickkids.ca (J.L.); cynthia.vandenhoven@sickkids.ca (C.V.); elise.heon@sickkids.ca (E.H.); ray.buncic@sickkids.ca (J.R.B.); 2 Department of Ophthalmology and Vision Sciences, University of Toronto, Toronto, ON M5T 3A9, Canada; twright@kensingtonhealth.org; 3 Kensington Eye Institute, Toronto, ON M5T 3A9, Canada; 4 INSERM, CNRS, Institut de la Vision, Sorbonne Université, 75012 Paris, France; isabelle.audo@inserm.fr (I.A.); christina.zeitz@inserm.fr (C.Z.); 5 CHNO des Quinze-Vingts, DHU Sight Restore, INSERM-DGOS CIC 1423, 75012 Paris, France; 6 Institute of Ophthalmology, University College of London, London EC1V 9EL, UK; 7 Program in Genetics and Genome Biology, The Hospital for Sick Children, Toronto, ON M5G 0A4, Canada; erika.tavares@sickkids.ca; 8 Department of Molecular Genetics, University of Toronto, Toronto, ON M5S 1A8, Canada; 9 Department of Genetic Counselling, The Hospital for Sick Children, Toronto, ON M5G 1X8, Canada

**Keywords:** *CACNA1F*, calcium channels/genetics, electroretinography, eye diseases, hereditary, myopia, night blindness/congenital, optic atrophy, retinal ganglion cells, retinal bipolar cells, tomography, optical coherence

## Abstract

Hemizygous pathogenic variants in *CACNA1F* lead to defective signal transmission from retinal photoreceptors to bipolar cells and cause incomplete congenital stationary night blindness in humans. Although the primary defect is at the terminal end of first-order neurons (photoreceptors), there is limited knowledge of higher-order neuronal changes (inner retinal) in this disorder. This study aimed to investigate inner retinal changes in *CACNA1F*-retinopathy by analyzing macular ganglion cell layer-inner plexiform layer (GCL-IPL) thickness and optic disc pallor in 22 subjects with molecularly confirmed *CACNA1F*-retinopathy. Detailed ocular phenotypic data including distance and color vision, refraction and electroretinogram (ERG) were collected. Distance vision was universally reduced (mean: 0.42 LogMAR), six had abnormal color vision and myopia was common (*n* = 15; mean: −6.32 diopters). Mean GCL-IPL thickness was significantly lower in patients (55.00 µm) compared to age-matched controls (*n* = 87; 84.57 µm; *p* << 0.001). The GCL-IPL thickness correlated with scotopic standard (*p* = 0.04) and bright-flash (*p* = 0.014) ERG b/a ratios and photopic b-wave amplitudes (*p* = 0.05). Twenty-one patients had some degree of disc pallor (bilateral in 19). Fifteen putative disease-causing, including five novel variants were identified. This study establishes macular inner retinal thinning and optic atrophy as characteristic features of *CACNA1F*-retinopathy, which are independent of myopia and could impact potential future treatment strategies.

## 1. Introduction

Congenital stationary night blindness (CSNB) is a group of genetically and phenotypically heterogeneous retinal disorders that follow autosomal dominant, autosomal recessive or X-linked patterns of inheritance and can be broadly categorized into those with a largely normal, or an abnormal, fundus appearance [1]. CSNB with a largely normal fundus can be classified based on electroretinogram (ERG) into the less common Riggs type representing rod phototransduction disruption [2], or the more common Schubert–Bornschein type [3] reflecting a post-phototransduction deficit in signal transmission between the photoreceptors and bipolar cells. In contrast, CSNB with an abnormal fundus comprises two distinct disorders: fundus albipunctatus and Oguchi disease, due to delayed retinoid recycling and defective deactivation of the rod phototransduction cascade, respectively [4,5,6].

Schubert–Bornschein CSNB is further subdivided into complete (cCSNB) and incomplete (iCSNB) phenotypes based on ERG patterns consistent with dysfunction of the ON- or both the ON- and OFF-bipolar pathways, respectively [7]. On scotopic (dark adapted; DA) standard and bright flash ERG (3.0 and 10 cd·s·m^−2^, respectively) stimulation, both cCSNB and iCSNB subjects demonstrate a normal a-wave with a markedly reduced b-wave resulting in an electronegative configuration [3]. On photopic (light adapted; LA) ERG testing, iCSNB subjects demonstrate markedly reduced a- and b-wave amplitudes to a standard flash (3.0 cd·s·m^−2^) and reduced 30 Hz flicker amplitudes, whereas cCSNB subjects exhibit subtle abnormalities to standard flash (broadened a-wave trough and a mildly reduced b-wave amplitude) and 30 Hz flicker (delayed and square-shaped trough) stimuli [1,8,9]. Both cCSNB and iCSNB show phenotype–genotype correlation, with genes involved in cCSNB encoding proteins mainly localized to post-synaptic retinal ON-bipolar cells, while genes responsible for iCSNB affect proteins predominantly expressed at the terminal end of the photoreceptor synapse, affecting both ON- and OFF-bipolar signaling [1].

Although incomplete CSNB may follow X-linked or autosomal recessive inheritance patterns, it is most commonly inherited as an X-linked condition due to mutations in *CACNA1F* [1]. Biallelic mutations in *CABP4* [10,11,12] and *CACNA2D4* [13] have been associated with autosomal recessive iCSNB, and infrequent *CACNA2D4* patients demonstrate slight pigment mottling at the fovea [13]. The ERG phenotype in *CACNA2D4*-related iCSNB is variable with some cases demonstrating preserved scotopic responses, and a reduced and delayed photopic standard flash ERG b-wave that has a multiphasic appearance [14]. Most recently, ERG findings compatible with iCSNB were also described in patients with biallelic loss-of-function variants in *RIMS2*. All *RIMS2* patients had optic disc pallor with most demonstrating retinal vascular attenuation; affected individuals also displayed neurodevelopmental disease and infrequently abnormal glucose homeostasis, making this a syndromic form of iCSNB [15]. Given the phenotypic variability within iCSNB and the fact that many patients complain of photophobia [10], rather than nyctalopia [1,16], “congenital rod-cone synaptic disorder” is a recently proposed term for the *CACNA1F*-related disorder [17].

Subjects with *CACNA1F*-related iCSNB commonly present with nystagmus, photophobia and/or nyctalopia, distance and color vision defects, variable myopic refractive error and essentially normal fundi apart from changes typical of high myopia (optic disc tilt, slight temporal disc pallor and fundus tessellation) [7,10]. There are rare reports of optic atrophy in association with *CACNA1F*-related retinopathy [18,19,20]. A child presenting with optic disc atrophy and retinal nerve fiber layer (RNFL) thinning was diagnosed as having *CACNA1F*-related iCSNB [18]. Further, an older Japanese sibship harboring a frameshift *CACNA1F* mutation demonstrated progressive vision loss with worsening optic atrophy and chorioretinal atrophy; it is unclear if the progressive neuro-retinopathy is *CACNA1F*-related [19]. A third study reported three *CACNA1F* patients described with normal-appearing fundi to have normal RNFL thickness but reduced macular combined ganglion cell layer plus inner plexiform layer (GCL-IPL) thickness compared to myopic controls [21].

Since *CACNA1F*-patients often have tilted discs due to high myopia, and nystagmus, reliable measurement of peripapillary RNFL thickness as an objective marker of optic atrophy can be challenging. There is, however, a fair correlation between RNFL and macular GCL-IPL thicknesses [22,23] and there is increasing use of both parameters to characterize optic neuropathies of varying etiologies [24,25,26,27,28]. In the present study, the authors measured macular GCL-IPL thickness and optic disc pallor in a cohort of *CACNA1F* subjects to assess if they had macular inner retinal thinning and optic disc atrophy in excess of the expected degree of disc pallor seen in myopia. In addition, peripapillary RNFL thickness measurements were analyzed when reliably measurable.

## 2. Materials and Methods

This retrospective study was approved by the institutional research ethics board at the Hospital for Sick Children, Toronto (REB no. 1000017804) and followed the tenets of the Declaration of Helsinki. Patients were identified from the hospital’s ocular genetics database and the records of 48 subjects with hemizygous mutations in *CACNA1F* (evaluated by CLIA-certified laboratories) were further analyzed. The pathogenicity of *CACNA1F*-variants was confirmed using ALAMUT visual software (https://www.interactive-biosoftware.com/alamut-visual/ (accessed on 18 February 2021)). Thirty-seven subjects had undergone optical coherence tomography (OCT) scanning, and of these, 22 had quantifiable macular OCT (see below for details), forming the study cohort. All subjects had ERG findings consistent with iCSNB. Limited clinical and genetic details of three subjects (case nos. 5, 6 and 16) have been reported previously (OCT descriptions were solely qualitative) [29,30].

Data collected included patient symptomatology, best-corrected visual acuity (BCVA) at distance in logMAR, cycloplegic refraction, color vision (Hardy Rand Rittler (HRR) Pseudoisochromatic Test, Good-Lite Co., Elgin, Illinois), contrast sensitivity (Pelli Robson Chart, Clement Clarke International, Essex, UK or Letter Contrast Sensitivity, M&S Technologies Inc., Niles, IL; which correlate well [31]), fundus photography, ERG (Diagnosys LLC, Lowell, MA), OCT and genetic results. Magnetic resonance imaging (MRI; *n* = 3) results were included where available.

**Figure 1:**
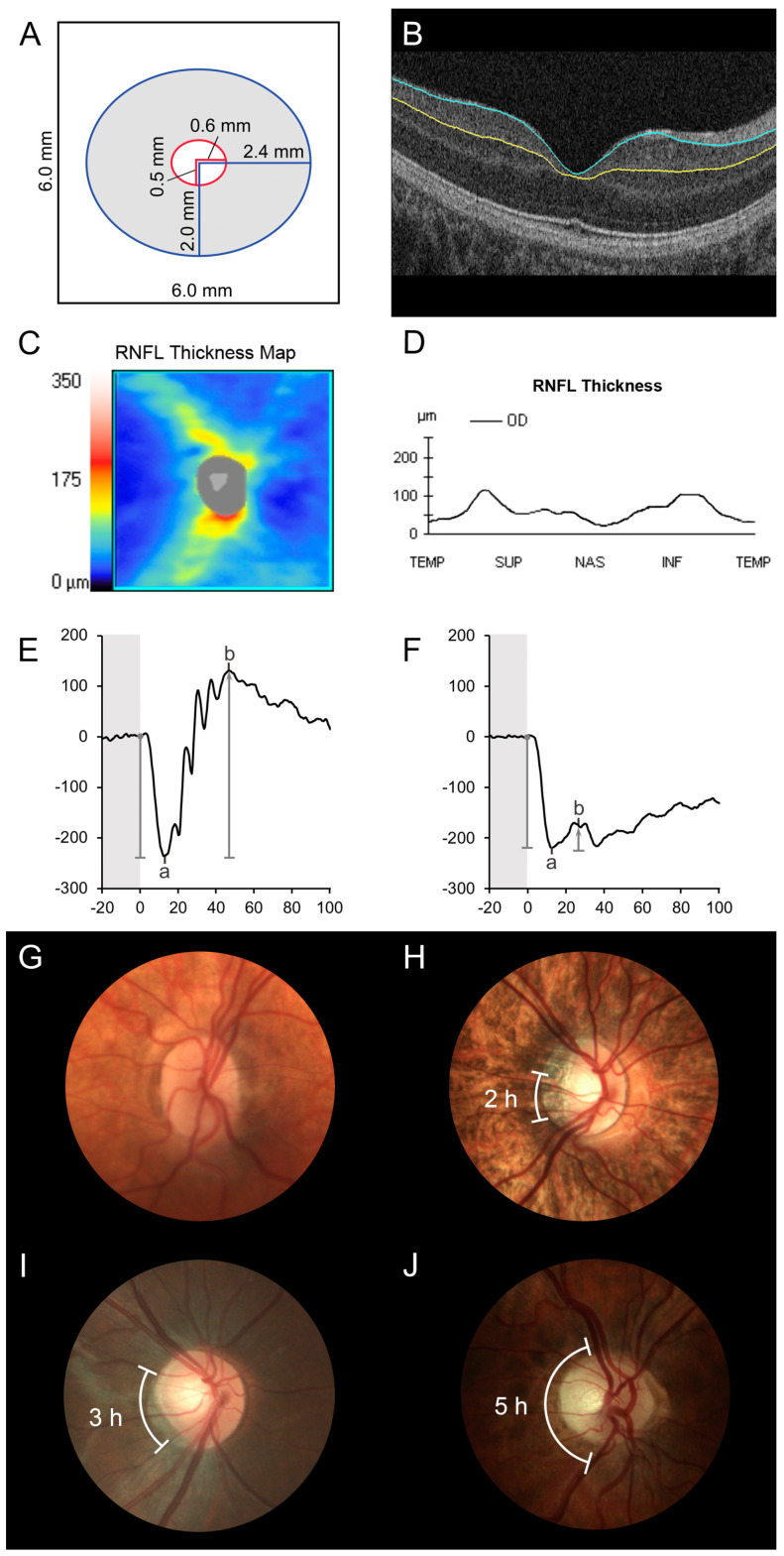
Optical coherence tomography (OCT) segmentation, electroretinogram (ERG) and disc photo grading. (**A**) Schematic diagram showing how average thickness for the sum of the ganglion cell and inner plexiform layers (GCL-IPL) was segmented from a 6 mm by 6 mm OCT macular cube scan for an elliptical annulus centered on the fovea with inner axis radius of 0.5 mm and outer axis radius of 2.0 mm, stretched by 20% in the horizontal direction. (**B**) Macular OCT segmentation: The blue line indicates the boundary between the GCL and retinal nerve fiber layer; the yellow line shows the boundary between the IPL and inner nuclear layer (case 16). (**C**,**D**) Peripapillary OCT segmentation: retinal nerve fiber layer (RNFL) thickness was determined using the Cirrus inbuilt analysis tool; (**C**) RNFL thickness map and (**D**) RNFL thickness graph (case 14; average RNFL thickness 61 µm; temporal RNFL thickness 44 µm). (**E**) Control ERG: The electroretinogram a-wave was measured from the prestimulus baseline to the trough, and the b-wave amplitude from the a-wave trough to the subsequent peak. (**F**) Electronegative ERG (case 9): the b-wave amplitude is smaller than the a-wave amplitude (b/a ratio < 1). (**G**–**J**) Color fundus photos were graded by a pediatric neuro-ophthalmologist as exhibiting (**G**) no disc pallor (case 10); (**H**) two clock hours of disc pallor (case 12); (**I**) three clock hours disc pallor (case 22) and (**J**) five clock hours of optic disc pallor (case 16).

Spectral-domain OCT was performed (Cirrus 4000, Carl Zeiss Meditec AG, Germany) using the macular cube protocol: 512 A-scans × 128 B-scans covering approximately 6 mm × 6 mm retina. All available macular cube scans for each patient were assessed for quality and those with the following suboptimal characteristics were excluded: (1) signal strength less than 6, (2) eye movement artifact within the central 80% of the scan area as seen in the en-face reflectance image, (3) area of data loss greater than 10% at the edge of the scan area or (4) presence of floater(s) obscuring macular area. All remaining OCTs were segmented using the IOWA Reference Algorithms v4.0.0 (Retinal Image Analysis Lab, Iowa Institute for Biomedical Imaging, Iowa City, IA) [32,33,34]. Average thickness for the sum of the GCL and IPL (GCL-IPL) was extracted using custom software [35,36] derived from an elliptical annulus centered on the fovea with inner axis radius of 0.5 mm and outer axis radius of 2.0 mm, stretched by 20% in the horizontal direction, corresponding to the Cirrus proprietary GCL-IPL analysis ring (Figure 1A,B). After segmentation, scans were reviewed for image quality and those with segmentation errors were excluded from analysis. Average GCL-IPL thickness from the most recent scan for each patient was selected. As there is a high incidence of refractive error, most often myopia, in patients with *CACNA1F*-retinopathy [10], comparative GCL-IPL thickness values were obtained using the protocol described above from a control group identified through our internal database (*n* = 87) with normal best-corrected vision of at least 0.10 logMAR and without any organic ocular pathology. The control population was subdivided into three groups based on the spherical equivalent (SE) of their refractive error: (1) emmetropia or hyperopia group: 40 eyes with SE ≥ 0 (mean SE + 0.86D), (2) low myopia group: 30 eyes with SE ≤ −0.5 and > −6.00D (mean SE − 3.22D) and (3) high myopia [37] group: 17 eyes with SE ≤ −6.00D (mean SE −8.38D). In addition, peripapillary RNFL thickness parameters derived from optic disc cube scans with good automatic segmentation were available in 6 of the 22 *CACNA1F* patients. A representative RNFL thickness analysis is shown in Figure 1C,D.

Full-field ERG data performed in accordance with the accepted International Society for Clinical Electrophysiology of Vision Standards were available in the study eye of 22 patients [38,39]. The amplitude of the a-wave (photoreceptor origin) was measured from the prestimulus baseline to the negative-peak or “trough”, and that of the b-wave (bipolar cell origin) was measured from the a-wave trough to the subsequent positive-peak (Figure 1E). The ratio of the b-wave to a-wave amplitude (b/a ratio) was calculated for the DA standard flash (3.0 or 2.29 cd·s·m^−2^), DA bright flash (10.0 or 7.6 cd·s·m^−2^) and LA standard flash (3.0 or 2.29 cd·s·m^−2^). In the earlier years, the lab used 2.29 and 7.6 cd·s·m^−2^ stimuli for standard and bright flash ERG but later switched to 3.0 and 10.0 cd·s·m^−2^, respectively [38,39]. A reduced b/a ratio denotes generalized bipolar cell dysfunction and a b-wave amplitude smaller than the a-wave amplitude (b/a ratio < 1) is termed an electronegative ERG (Figure 1F).

True-color optic disc photos, available for 21 patients, were qualitatively evaluated by an experienced pediatric neuro-ophthalmologist noting the presence or absence of pallor of the disc substance as reflected in the neuroretinal rim. The extent of pallor was documented according to the number of clock-hours of the disc, which appeared pale, as was the presence or absence of a peripapillary crescent [40].

Data from one eye per patient was included in the study analysis. OCT and all ophthalmic data were collected from the same eye; the right eye was selected (*n* = 17) when the macular OCT was of sufficient quality and the left eye was included otherwise (*n* = 5).

Statistical analyses were performed using R version 3.6.2 (R Foundation for Statistical Computing, Vienna, Austria). Analysis of variance (ANOVA) was used to compare the differences among group means with post-hoc Tukey test to determine statistical significance. A two-sample *t*-test was used to compare subject age between patients and controls, and to compare GCL-IPL thickness between the groups of patients with, and without, RNFL measurements. Linear regression was used to evaluate the effect of SE on GCL-IPL thickness and on ERG parameters, the interaction between these parameters in the presence of SE was examined using two-way ANOVA. A *p* value of ≤0.05 was taken to be statistically significant.

## 3. Results

### 3.1. Patient Demographics and Clinical Features

Twenty-two males with molecularly confirmed hemizygous mutations in *CACNA1F* were included in the study. The average age of the patient cohort was 14.3 years (range 6–58 years). Nystagmus occurred in 50% (7 of 14) of patients for whom its presence or absence was documented. Similarly, nyctalopia and photophobia were reported in 50% (8 of 16) and 44% (7 of 16) of documented cases, respectively. The BCVA was reduced in all patients (mean 0.42 logMAR, median 0.40 and range 0.10–0.80 logMAR). The mean SE refractive error was −6.32D (median −7.38 and range −20.50 to + 2.50D), with myopia being common (68%; 15 of 22). Color vision was normal in the majority (71%; 15 of 21), whereas six patients showed red-green color defects, which were classified as mild (*n* = 4), medium (*n* = 1) or strong (*n* = 1) on HRR. Only 23% (5 of 22) *CACNA1F*-subjects had normal contrast sensitivity; the mean contrast sensitivity was 1.36 log units (range 0.90–1.80 log units) (Table 1). Two of the three patients who had MRI to evaluate for optic atrophy were found to have unremarkable optic nerves (cases 11 and 14) and one displayed bilateral, mild changes compatible with optic nerve atrophy (case 16).

### 3.2. Optical Coherence Tomography

**Figure 2:**
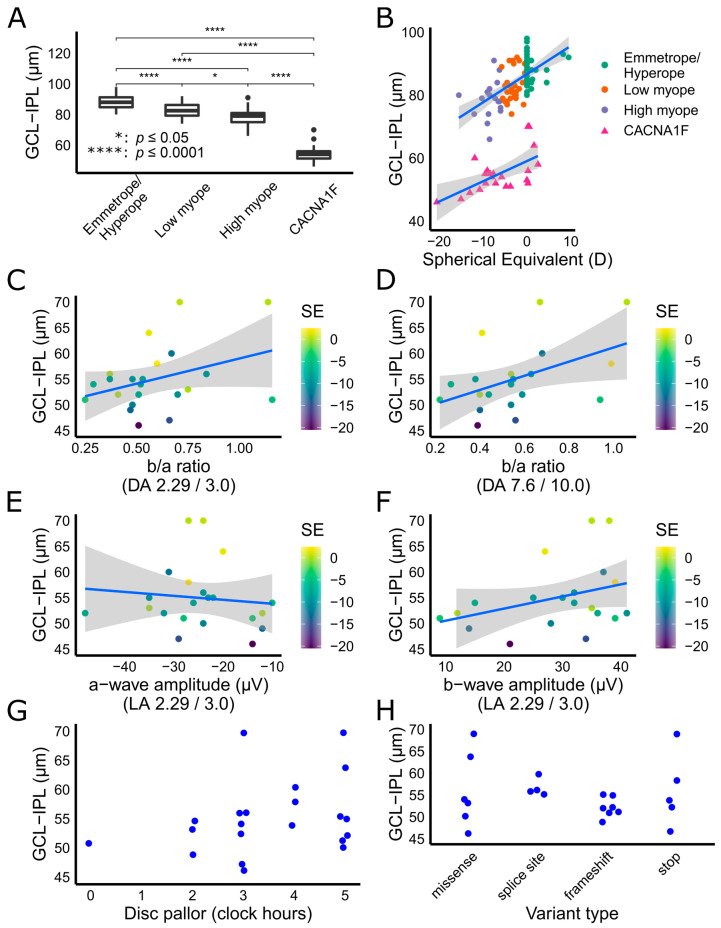
Macular ganglion cell layer plus inner plexiform layer (GCL-IPL) thickness in *CACNA1F* retinopathy. (**A**) The GCL-IPL in *CACNA1F* patients was significantly thinner than the emmetropia or hyperopia, low myopia and high myopia control groups. (**B**) Scatter plot showing average GCL-IPL thickness from individual *CACNA1F* patients and control subjects. For every diopter decrease in spherical equivalent there was a decrease in GCL-IPL thickness, which was not significantly different between *CACNA1F* patients and controls. (**C**,**D**) There was a statistically significant correlation between lower b/a ratio and reduced GCL-IPL thickness for dark-adapted (DA) standard flash (2.29 or 3.0 cd·s·m^−2^) and DA bright flash (7.6 or 10.0 cd·s·m^−2^) electroretinograms. (**E**) The light adapted (LA) standard flash a-wave amplitude did not correlate with GCL-IPL thickness, however, (**F**) a smaller LA standard flash b-wave amplitude correlated with a lower GCL-IPL thickness. (**G**,**H**) There was no correlation between GCL-IPL thickness and extent of disc pallor in clock hours (**G**) or variant type (**H**).

The macular GCL-IPL thickness values in *CACNA1F*-patients (*n* = 22) were compared to controls (*n* = 87). Average age in the control population was 12.0 years (range 4–53 years), which did not differ significantly from the patient cohort (*p* = 0.337). The mean (SD) GCL-IPL thickness in the *CACNA1F* group was 55.00 (6.17) µm, which was significantly thinner compared to controls (84.57 (6.15) µm; F_(3.105)_ = 199; *p* << 0.001). Post-hoc Tukey tests indicate GCL-IPL thickness in the *CACNA1F* group was significantly thinner than in the emmetropia or hyperopia (88.25 (4.52) µm; *p* << 0.001), low myopia (83.07 (4.94) µm; *p* << 0.001) and high myopia (78.59 (5.98) µm; *p* << 0.001) control subgroups (Figure 2A).

For every diopter decrease in spherical equivalent there was a decrease in GCL-IPL thickness of 0.81 µm, (R^2^_adjusted_ = 0.86, *p* << 0.01), which was not significantly different between *CACNA1F*-iCSNB and control subgroups (Figure 2B). After correcting for refractive error, however, macular GCL-IPL thickness in the CSNB group was on average thinner by 26.37 µm compared to the control group (F_(2.106)_ = 326, *p* << 0.001).

As the majority of the study (21/22) and control (85/87) subjects were under 25 years of age, we excluded the three older subjects from any analysis including age as a variable. After the exclusion of one *CACNA1F*-subject with age > 25 years (case 6, 58 years) and 2 control subjects (aged 34 and 53 years), age was negatively correlated with GCL-IPL thickness in both CSNB and normal controls (R^2^_adjusted_ = 0.79, *p* = 0.03) as previously reported [47,48,49,50]; however, the rate of thinning was not significantly different between the groups (*p* = 0.15).

Six patients in the *CACNA1F* cohort (mean age 12.5, range: 6–17 years) had good quality optic disc cube scans, and RNFL thickness was determined using the Cirrus inbuilt analysis tool (Table 2). Mean (SD) average peripapillary RNFL thickness was 68.67 (4.72) µm and mean (SD) temporal RNFL thickness was 51.33 (13.81) µm. Both mean values were lower than similar populations reported in literature (Table 3) [51,52,53,54,55,56,57]. The average RNFL values for all six *CACNA1F* patients were below the lower range reported in normal children, and two of four myopic patients had values outside the lower range reported for normal myopes. The temporal RNFL measures were below the lower range or 5th percentile for pediatric and myopic populations in four of six study subjects. The mean (SD) average macular GCL-IPL thickness for these six iCSNB subjects was 54.50 (3.67) µm, which was similar to the rest of the cohort (55.19 (7.16) µm; *p* = 0.77). The OCT values for each study eye are shown in Table 2.

### 3.3. Full-Field Electroretinogram Results

The DA standard flash ERG showed an electronegative configuration in 20 of 22 patients (mean (SD) b/a ratio: 0.59 (0.23); range 0.25–1.16). The DA bright flash ERG was electronegative in all except one tested (mean (SD) b/a ratio: 0.56 (0.22); range 0.22–1.06 (*n* = 20)). The LA standard flash ERG was electronegative in only three patients (mean (SD) b/a ratio: 1.18 (0.25); range 0.64–1.58 (*n* = 21)).

After controlling for SE, the macular GCL-IPL thickness was weakly correlated with the DA standard flash b/a ratio (R^2^_adjusted_ = 0.41, *p* = 0.04; Figure 2C), DA bright flash b/a ratio (R^2^_adjusted_ = 0.45, *p* = 0.014; Figure 2D) and LA standard flash b-wave amplitude (R^2^_adjusted_ = 0.41, *p* = 0.05; Figure 2F). The correlation with GCL-IPL thickness did not reach significance for LA standard flash b/a ratio (R^2^_adjusted_ = 0.42, *p* = 0.08) or LA standard flash a-wave amplitude (R^2^_adjusted_ = 0.34, *p* = 0.56; Figure 2E). Individual values for b/a ratio and amplitude measurements are shown in Table 2.

### 3.4. Optic Disc Evaluation and Fundus Findings

Twenty-one patients had color fundus photos available; 19 had some degree of disc pallor in both eyes (measured in observable clock hours) and the remaining 2 had unilateral disc pallor (Table 2). In the study eye, one disc (5%) was graded as having no pallor, three discs (14%) with two clock hours of pallor, seven (33%) with three clock hours, three (14%) with four clock hours and seven (33%) with five clock hours of pallor. Representative disc photos from four subjects are shown in Figure 1G–J. There was no significant correlation between macular GCL-IPL thickness and number of clock hours of disc pallor (R^2^_adjusted_ = 0.03, *p* = 0.22; Figure 2G).

Fifteen subjects showed myopic retinal changes (bilateral fundus tessellation (*n* = 14), white without pressure (*n* = 4), posterior staphyloma (*n* = 2), peripheral degeneration (*n* = 2) and/or unilateral Forster–Fuchs spot (*n* = 1)). Four cases displayed normal fundi and five had hypopigmented fundi more typical of Åland island eye disease, a phenotypic variant of *CACNA1F* iCSNB [20,29,59]; two in the latter group also had myopic changes. Foveal hypoplasia was identified in six cases on fundus photographs or OCT; the grading [58] is shown in Table 2.

### 3.5. Genetic Results

Fifteen putative disease-causing variants were identified in the study (Table 1); these included missense (*n* = 5, 6 subjects), nonsense (*n* = 4, 5 subjects), frameshift (*n* = 3, 7 subjects), splice site (*n* = 2, 3 subjects) and silent variants predicted to affect splicing (*n* = 1, 1 subject). Five of the variants are novel, which were p.(Gln561*), p.(Val611Glyfs*32), p.(Gln1157Gln), c.3741+2T>C and p.(Pro1492Ser). The details of the novel variants, including the predicted pathogenicity, conservation scores and ACMG classification are summarized in Table 4. None have previously been identified in large population databases (gnomAD) [60]. Both p.(Gln561*) and p.(Val611Glyfs*32) are expected to produce truncated protein or the mRNA produced are likely to be targeted for nonsense-mediated decay. The silent variant p.(Gln1157Gln) affects the last nucleotide of exon 28, is predicted to alter the natural splice donor site 1 base pair downstream and is thereby likely to cause exon 28 skipping. The c.3741+2T>C substitution is located on the canonical donor splice site of intron 30, and is expected to cause exon 30 skipping. The p.(Pro1492Ser) variant affects a highly conserved residue and is predicted pathogenic by all in silico tools; further, there are other substitutions at and near position 1492 (p.(Pro1492Ala) and p.(Gly1494Arg)) that have been reported to cause iCSNB [1,42]. The mutation segregated with the disease phenotype in all tested cases (*n* = 18); among the four cases where segregation analysis was unavailable, two harbored previously reported mutations (case 19: p.(Pro1492Ala) [1,42] and case 21: p.(Arg1502*) [42]) and two harbored novel disease-causing variants (case 9: p.(Val611Glyfs*32) and case 17: p.(Gln1157Gln)). The macular GCL-IPL thickness did not differ regardless of the type of mutation ((F_(3,18)_ = 0.68, *p* = 0.57; Figure 2H)). 

## 4. Discussion

This is the first comprehensive study to have reported marked macular GCL-IPL thinning in subjects with molecularly confirmed *CACNA1F*-related iCSNB. All subjects in the current cohort demonstrated at least some degree of optic disc pallor in at least one eye. The majority of patients were myopic and the peripapillary RNFL thickness was reduced below average in all six tested individuals. Previously, only rare instances of optic nerve pallor or atrophy had been reported in *CACNA1F*-retinopathy [18,19,20] and since many *CACNA1F* subjects have high myopia [10], this has been proposed to account for the optic disc pallor, often at the temporal disc, in these patients [7,72,73]. The present study identified disc pallor together with thinner GCL-IPL and RNFL compared to a myopic control cohort and values reported in the literature suggesting that both findings are unrelated to myopia, and the optic disc pallor in *CACNA1F*-patients is due to atrophy. Our findings also indicate that inner retinal and optic disc changes are far more common than originally thought in this condition, traditionally described as having a normal fundus.

The incidence of nystagmus (50%), nyctalopia (50%) and photophobia (44%) in our cohort was similar to those identified in two large studies of *CACNA1F*-related iCSNB, which documented rates of 44–65%, 58–60% and 50%, respectively [10,73]. The BCVA was reduced in all study subjects. The mean BCVA in this study (0.42 LogMAR) was similar to that reported by Allen et al. (0.40 LogMAR) [74] and the range of BCVA in our cohort (0.10–0.80) was similar to two previous studies, which had reported 0.10–1.00 and 0.10–1.30 LogMAR, respectively [10,73]. Myopic refractive error was common in the current cohort (73%), as reported in other series in literature (81%–85%) [10,73]. Only red-green color defects were observed in the current study (29%, HRR testing); a previous group reported color vision defects in 46% (21/46, using one of D15, Ishihara or HRR) of their cohort [10]. Only a small proportion of patients in either study had strong color vision defects (5% in the current study vs. 13% in Bijveld et al.) [10], however, a different study reported strong color defects in all tested family members (*n* = 6; D15 and Ishihara) [75].

The average macular GCL-IPL thickness was severely reduced in our *CACNA1F* cohort compared to controls with high myopia (*p* << 0.001). Further, most tested subjects had thinner average RNFL compared to myopic [55,56,57] or pediatric control cohorts [51,52,53,54]. A previous study suggested thinner GCL-IPL and normal RNFL thickness in three cases of *CACNA1F*-related iCSNB, although thickness measurements were not reported [21]. Further, selective inner retinal thinning, as evidenced by reduced average GCL-IPL thickness (range 59–65 μm), was reported in three patients with *GRM6*-related cCSNB; the authors hypothesized reduced bipolar and/or ganglion cell numbers or altered inner retinal synaptic structure to be the cause [76]. It is notable that average GCL-IPL thicknesses in our study are similar (55 (6.17) μm; range: 46–70 μm) to those reported in *GRM6* cCSNB patients. Additionally, of note are various mouse models for iCSNB (*Cacna1f*, *Cabp4* and *Cacna2d4*) primarily demonstrating abnormal synapses and thinning in the outer plexiform layer with no apparent changes in the inner retina including the GCL and IPL [77,78,79]. This may suggest that inner retinal changes are perhaps unique to human *CACNA1F*-phenotype in comparison to known animal models of the disease. One of the patients in the present cohort was additionally observed to have abnormal synapses in the outer retina [30].

The DA standard and bright-flash ERGs were electronegative in the majority of subjects in the current study with mean b/a ratios of 0.59 and 0.56, respectively, indicative of severe generalized rod ON-bipolar cell dysfunction. An electronegative ERG (or reduced b/a ratio) to DA standard flash or higher intensities is a characteristic feature of *CACNA1F* (median b/a ratio: 0.70) [10] and other forms of CSNB [1,10,73,74]. The mean LA standard flash ERG b/a ratio was 1.18 in our cohort; a similar average b/a ratio of 1.35 in the cohort reported by Bradshaw et al. was significantly reduced compared to controls (2.31) [80], indicative of generalized cone ON- and OFF-bipolar cell dysfunction. In the present study, GCL-IPL thickness showed weak correlation with DA standard flash and bright flash ERG b/a ratios, and LA standard flash b-wave amplitudes, with thinner GCL-IPL corresponding to lower b/a ratios or b-wave amplitudes, respectively. These results are novel and might indicate some superseding inner retinal (bipolar cell) dysfunction in addition to the signal transmission defect at the terminal end of photoreceptors in *CACNA1F* disorder. Future studies, however, are needed to further validate this observation.

All subjects in the current cohort had at least 2 clock hours of optic disc pallor in at least one eye and most had bilateral disc pallor. To date, a few cases in the literature have documented optic disc pallor or atrophy in *CACNA1F*-related iCSNB [18,19,20] and these reports are limited to the clinical appearance, rather than the graded measurement of optic disc pallor or ganglion cell structural integrity. All seven reported cases of iCSNB associated with *RIMS2*, a regulator of synaptic membrane exocytosis localized to rod photoreceptors and the outer plexiform layer, from 3 unrelated, ethnically diverse families demonstrated clinically appreciable optic disc pallor [15]. Inner retinal thinning was seen in the three subjects for whom OCT was available, and RNFL thinning was shown in two of them [15]. Whilst the authors advise optic disc and inner retinal changes should be interpreted with caution in the context of myopia, it is noted that none of the cases had high myopia (range: +6.00 to −4.50 D; only three were myopic) [15]. In 1983 Heckenlively et al. reported optic disc anomalies (interpreted as representing atrophy, dysplasia, or both) in subjects with CSNB; five had tilted discs with a lack of visible temporal disc tissue, two had dysplastic nerves and three had disc pallor without tilt [81]. Further, we showed that inner retinal thinning in *CACNA1F*-related iCSNB patients is in excess of controls with the same degree of myopia, strongly suggesting the possibility that optic pallor is consistent with optic atrophy, which is likely overlooked in *CACNA1F*-related iCSNB as a whole. Optic disc pallor has not been described in cases of iCSNB due to *CABP4* or *CACNA2D4* mutations [13,14]. The clock hours of appreciable disc pallor did not correlate with GCL-IPL thickness. This could in part be attributed to clinical difficulty in fundoscopy in identifying optic atrophy in the presence of significant myopia because of a prominent white scleral crescent and very little visible temporal disc tissue in the presence of a very obliquely exiting optic nerve (tilted disc) in many patients [82,83,84]. OCT measures of RNFL atrophy and decreased GCL-IPL thickness, on the other hand, are objective markers of axonal atrophy and support the presence of nerve atrophy within the disc in this study cohort.

*CACNA1F* encodes the primary subunit (α_1f_) of the L-type voltage-gated calcium channel, a 1977 amino acid protein with four homologous transmembrane domains flanked by intracellular components (N and C termini) [85]. Fifteen putative disease-causing variants including missense (33%), nonsense (27%), frameshift (20%) and splice-site (20%) were identified in our cohort. A previous study with 26 pathogenic mutations reported a higher proportion of nonsense and frameshift variants (27% each) followed by missense variants (23%). Amongst the five novel mutations, two each were nonsense (p.(Gln561*) and p.(Val611Glyfs*32)) and splice-site ((c.3741+2T>C) and p.(Gln1157Gln)) variants. Both Gln561 and Val611 are in the second transmembrane domain and a premature stop codon will lead to severely truncated protein missing the 3rd and 4th transmembrane domains and the C termini. Further, the mRNA produced is likely removed by nonsense mediated decay. The synonymous variant (c.4474C>T; p.(Gln1157Gln)) affects the last nucleotide in exon 28 and is predicted to alter the splice donor site. Recently, two other exonic synonymous variants in *CACNA1F* (c.646C>T; p.(Leu216Leu) and c.1719G>A; p.(Thr573Thr)) were reported to affect splicing using a minigene approach [42]. In addition, there are reported instances of pathogenic synonymous exonic variants affecting the last nucleotide of an exon leading to a splice site defect in other disorders [86,87]. Although the average macular GCL-IPL thickness in the *CACNA1F*-subjects was reduced regardless of the mutation class, there appeared to be variability within each mutation class (Figure 2F). This is perhaps not surprising as others have shown considerable intra- and inter-familial variability in phenotypic features (including refractive error, visual acuity and dark adaptation thresholds) in subjects harboring the same *CACNA1F* mutation [10,73].

The *Cacna1f* gene is well expressed in wildtype mouse and rat retina; whilst most studies confirm its expression at the photoreceptor terminal in the outer plexiform layer [45,88,89,90,91,92,93], some report expression in the inner part of inner nuclear layer and GCL [20,45,90,91]. Further, there is electrophysiological evidence from *Cacna1f*-deficient mouse lines to suggest that this subunit contributes to calcium influx in the retinal bipolar cells (Qi Lu PhD dissertation, Wayne State University—referenced in [85]). Hence the findings of GCL-IPL thinning and optic disc pallor observed in the current study may either be due to defective *CACNA1F* function in the inner retinal layers or perhaps even due to transsynaptic changes hypothesized by some in CSNB [76,81]. In either case, our findings are important in the context of novel approaches under development to treat *CACNA1F*-retinopathy [94], as it is likely that inner retinal changes contribute to the visual deficit in the disorder in humans and as such, treatment targeted at a defect in the photoreceptor synapse alone may not be sufficient to restore vision.

To summarize, this study identified inner retinal thinning and optic atrophy as characteristic features of iCSNB due to hemizygous pathogenic mutations in *CACNA1F*. The inner retinal thinning was independent of myopia and mutation type; although there was variability in the presence of myopia in our cohort, GCL-IPL thickness was uniformly reduced, and thinner than a myopic control population. The macular OCT was useful to objectively differentiate optic atrophy from disc pallor alone, such as that associated with high myopia. A prospective natural history study would help identify whether these inner retinal changes are stationary or progressive in nature.

## Figures and Tables

**Table 1:** Characteristics of the study population.

Case	Age, y	NM_005183.3 cDNA and AA Variations	VA, OD; OS	Refraction, OD; OS	Color, HRR, OU	CS, OD; OS	Symptoms		
							Nystagmus	Nyctalopia	Photophobia
1	10	c.245G>A; p.(Arg82Gln) [1]	0.40; 0.40	+1.00/+1.25; +2.00/+0.75	mild RG	1.30; 1.30	-	-	-
2	15	c.1047G>A; p.(Trp349*) [1,41]	0.50; 0.50	−9.25/+4.25; −9.50/+4.00	normal	1.40; 1.50	-	-	+
3 ^a^	14	c.1681C>T; p.(Gln561*) ^N^	0.20; 0.20	−9.75/+2.50; −8.75/+1.50	normal	1.50; 1.50		-	-
4 ^a^	16	c.1681C>T; p.(Gln561*) ^N^	0.50; 0.60	−15.75/+1.50; −14.75/+1.25	normal	1.30; 1.20	-	+	-
5 ^b^	11	c.1807G>C; p.(Gly603Arg) [29]	0.40; 0.48	−12.25/+3.00; −12.25/+3.00	strong RG	1.50; 1.50		+	+
6 ^b^	58	c.1807G>C; p.(Gly603Arg)	0.40; 0.40	plano; plano	mild RG	1.65; 1.65		+	
7	9	c.1832del; p.(Val611Glyfs*32) ^N^	0.48; 0.60	−12.50/+4.00; −11.50/+4.00	normal	1.40; 1.50	+	+	+
8	14	c.1849C>T; p.(Gln617*) [1]	0.30; 0.40	+1.00/+3.00; +1.00/+3.00	normal	1.30; 1.30	+	+	+
9	6	c.2094_2095del; p.(Gln699Glyfs*12) [42]	0.80; 0.80	−7.50/+2.50; −5.50/+3.50	mild RG	1.20; 1.20		-	-
10	8	c.2576+1G>A; p.? [42,43]	0.30; 0.48	−0.25/+1.00; −1.00/+0.75	normal	1.20; 1.20		-	+
11	9	c.2576+1G>A; p.?	0.80; 0.90	−9.00; −9.75		0.90; 0.90	-	+	-
12	8	c.3166dup; p.(Leu1056Profs*11) [1,44,45]	0.40; 0.40	−14.00/+2.00; −14.00/+2.75	normal	1.50; 1.50	+		-
13 ^c^	13	c.3166dup; p.(Leu1056Profs*11)	0.40; 0.30	−9.25/+3.25; −9.50/+2.75	normal	1.40; 1.40	-		
14 ^c^	13	c.3166dup; p.(Leu1056Profs*11)	0.48; 0.60	−11.00/+3.25; −10.00/+3.25	mild RG	1.30; 1.20	+		
15 ^c^	16	c.3166dup; p.(Leu1056Profs*11)	0.30; 0.30	−7.25/+5.00; −6.75/+4.50	normal	1.40; 1.40			
16	17	c.3166dup; p.(Leu1056Profs*11)	0.18; 0.18	+0.25; +0.25	medium RG	1.40; 1.30	+	+	+
17	9	c.3471G>A; p.(Gln1157Gln) ^N, S^	0.40; 0.40	−14.25/+4.75; −14.25/+4.75	normal	1.30; 1.30	-		
18	17	c.3741+2T>C; p.? ^N^	0.40; 0.40	−10.25/+2.75; −9.75/+2.75	normal	1.30; 1.30		+	-
19	20	c.4474C>G; p.(Pro1492Ala) [1,46]	0.48; 0.30	−21.75/+4.00; −22.75/+4.50	normal	1.30; 1.50		-	-
20	16	c.4474C>T; p.(Pro1492Ser) ^N^	0.48; 0.30	−6.75/+2.00; −6.50/+2.00	normal	1.30; 1.30	-	-	+
21	7	c.4504C>T; p.(Arg1502*) [42]	0.54; 0.54	−1.50/+3.50; −3.00/+4.00	normal	1.10; 1.10	+		
22	10	c.5156G>T; p.(Arg1719Met) [42]	0.10; 0.10	−1.00/+3.00; −2.00/+3.00	normal	1.80; 1.80	+	-	-

^a^, family 1; ^b^, family 2; ^c^, family 3; y, years; AA, amino acid; ^N^, novel variant; ^S^, Silent mutation affecting the last nucleotide of the exon and predicted to affect splicing; VA, visual acuity in LogMAR; OD, right eye; OS, left eye; D, diopters; HRR, Hardy Rand Rittler; OU, both eyes; RG, red-green; CS, contrast sensitivity in log units.

**Table 2:** Optical coherence tomography and electroretinogram parameters, and optic disc pallor grading.

Case	Study Eye	Average Macular GCL-IPL Thickness, µm	Average (Temporal) RNFL Thickness, µm	ERG b/a Ratio DA Standard Flash	ERG b/a Ratio DA Bright Flash	ERG a-Wave; b-Wave amplitude (µV) LA Standard Flash	ERG b/a Ratio LA Standard Flash	Disc Pallor, OD; OS, h (Description)	Fundus Findings, OU
1	OD	64		0.56	0.41	–20; 27	1.35	5; 4	Normal
2	OD	52		0.51	0.44	–48; 36	0.75	3 (PC); 4 (PC)	Tessellated, FH (1)
3	OS	54		0.52	0.54	–26; 32	1.23	5 (PC); 4 (PC)	Tessellated, WWP
4	OD	47		0.66	0.56	–29; 34	1.17	3 (PC); 3 (PC)	Tessellated, WWP, staphyloma, FH (1)
5	OD	50		0.48	0.54	–24; 28	1.17	5 (PC); 5 (PC)	Blonde, FH (2)
6	OD	53		0.75		–35; 35	1.00	2; 2	Normal
7	OS	55		0.48		–35; 25	0.71	5; 5	Tessellated
8	OD	58	68 (40)	0.60	0.99	–27; 39	1.44	4; 4	Peripheral atrophy
9	OS	51	66 (51)	0.25	0.22	–14; 9	0.64	3; 0	Appearance of sheathing around superotemporal vascular arcades
10	OD	56		0.37	0.54			3; 0	Tessellated, blonde, FH (1)
11	OD	56		0.84	0.63	–24; 32	1.33	3 (PC); 3 (PC)	Blonde
12	OD	49		0.47	0.40	–12; 14	1.17	2 (PC); 4 (PC)	Tessellated, WWP
13	OD	55		0.53	0.55	–22; 30	1.07	5 (PC); 3 (PC)	Tessellated
14	OD	52	61 (44)	0.70	0.59	–32; 41	1.28		Tessellated, lattice degeneration
15	OD	51		1.16	0.94	–28; 39	1.39	5 (PC); 5 (PC)	Tessellated, FH (1)
16	OD	52	72 (39)	0.41	0.40	–12; 12	1.00	5 (PC); 5 (PC)	Tessellated
17	OD	60	71 (75)	0.67	0.68	–31; 37	1.19	4 (PC); 5 (PC)	Tessellated
18	OS	55		0.37	0.38	–23; 25	1.09	3 (PC); 2	Tessellated, WWP
19	OS	46		0.51	0.39	–14; 21	1.50	2 (PC); 3 (PC)	Tessellated, blonde, staphyloma. Fuchs spot (OD)
20	OD	54	74 (59)	0.29	0.27	–10; 15	1.50	3 (PC); 4	Tessellated, blonde, FH (2)
21	OD	70		1.14	1.06	–27; 35	1.30	5; 4	Normal
22	OD	70		0.71	0.67	–24; 38	1.58	3; 3	Appearance of perivascular sheathing along some vessels

OD, right eye; OS, left eye; GCL-IPL, ganglion cell layer and inner plexiform layer; RNFL, retinal nerve fiber layer; ERG, electroretinogram; DA, dark-adapted; LA, light-adapted; h, clock hours; PC, peripapillary crescent; OU, both eyes; FH, foveal hypoplasia (optical coherence tomography grade [58]); WWP, white-without-pressure. GCL-IPL thickness, RNFL thickness and ERG b/a ratios are shown for the study eye; the right eye was selected when macular optical coherence tomography was of sufficient quality.

**Table 3:** Reported retinal nerve fiber layer thickness in children and myopes.

Source	Age, y	n	Refraction, SE (D)	Average RNFL (mm)	Temporal RNFL
Guragac et al., 2017 [51]	3–17 ^a^	318	–4.00 to +3.00 ^a^	96.49 (10.10) ^b^; 80.00–114.00 ^d^	67.60 (9.93) ^b^; 53.95–87.00 ^d^
Al-Haddad et al., 2014 [52]	6–17 ^a^	108	–4.25 to +5.00 ^a^	95.6 (8.7) ^b^; 80–111 ^d^	66.4 (8.9) ^b^; 54–84 ^d^
Barrio-Barrio et al., 2013 [53]	4–17 ^a^	283	–4.88 to +5.25 ^a^	97.40 (9) ^b^; 77.0–121.7 ^a^; 82.4–113.3 ^d^	67.4 ^b^; 51.8–83.3 ^d^
Elia et al., 2012 [54]	6–14 ^a^	344	–2.50 to +6.25 ^a^	98.46 (10.79) ^b^	69.35 (11.28) ^b^
Lee et al., 2020 [55]	39.3 (12.2) ^b^	80	–6.50 (–8.50 to –4.25) ^c^	89.88 (8.87) ^b^	
Biswas et al., 2016 [56]	36.0 (12.3) ^b^	180	–17.1 to –6.0D ^a^	89.6 (7.2) ^b^; 70–110 ^a^	
Hwang et al., 2012 [57]	19–25 ^a^	255	–11.00 to 0.00 ^a^	97.14 (6.63) ^b^; 72–118 ^a^	74.03 (11.13) ^b^; 53–105 ^a^
Present study	6–17 ^a^	6	–11.88 to +2.50^a^	68.67 (4.72) ^b^; 61–74 ^a^	51.33 (13.81) ^b^; 39–75 ^a^; 39.25–71.00 ^d^

y, years; SE, spherical equivalent; D, diopters; RNFL, retinal nerve fiber layer; ^a^ range; ^b^ mean (SD); ^c^ median (interquartile range); ^d^ 5th–95th percentile.

**Table 4:** Novel *CACNA1F* variants: pathogenicity and conservation scores, allele frequency in gnomAD and ACMG classification.

Genomic Position (hg19, chr X)	NM_005183.3—cDNA; Protein Position	Predicted Effect	Splicing or Pathogenicity Scores	GnomAD [60] (v2.1.1)	PhyloP [61]	ACMG Criteria; Classification [62]
g.49082374G>A	c.1681C>T; p.(Gln561*)	nonsense	N/A	N/A	4.73	PVS1, PM2, PP1; pathogenic
g.49081301del	c.1832del; p.(Val611Glyfs*32)	frameshift stop	N/A	N/A	3.76	PVS1, PM2; likely pathogenic
g.49071802C>T	c.3471G>A; p.(Gln1157Gln)	splicing, predicted loss of donor site 1 bp downstream	MaxEntScan [63]: −100.0%, NNSPLICE [64]: −95.8%, SSF [65]: −15.7%, spliceAI [66]: 0.96	N/A	2.47	PM2, PP3; uncertain significance
g.49070617A>G	c.3741+2T>C; p.?	splicing, predicted loss of donor site 2 bp upstream	MaxEntScan [63]: −100.0%, NNSPLICE [64]: 0.0%, SSF [65]: −13.8%, spliceAI [66]: 0.97	N/A	4.08	PVS1, PM2, PP1, PP3; pathogenic
g.49067094G>A	c.4474C>T; p.(Pro1492Ser)	missense	SIFT [67]: 0 (deleterious), Poly Phen-2 [68]: 1 (PD), Align GVGD [69]: C65, MutationTaster [70]: 1(Del), Revel [71]: 0.83(Del)	N/A	5.45	PM1, PM2, PM5, PP3; likely pathogenic

Ref. [60] Genome Aggregation Database (gnomAD, https://gnomad.broadinstitute.org/ (accessed on 18 February 2021); population frequency across 141,456 human genomes); [61] Phylogenetic *p* value vertebrates (PhyloP, evolutionary conservation at individual alignment sites. Positive values mean more conservation that expected by neutral evolution, values > 4 are extremely conserved) [63]; ACMG, American College of Medical Genetics and Genomics. Under the AMCG guidelines, each pathogenic criterion is weighted as very strong (PVS1), strong (PS1-4), moderate (PM1-6), or supporting (PP1-5); Maximum Entropy Scan (MaxEntScan, This method is based on the ‘Maximum Entropy Principle’ and generalizes most previous probabilistic models of sequence motifs such as weight matrix models and inhomogeneous Markov models, −100% means complete loss of splicing score for a site), [64] Neural Network Splice algorithm with k-fold cross-validation (NNSPLICE, https://github.com/AnastasiiaInna/neural-network-splice-algorithm (accessed on 18 February 2021)), [65] Splicing Sequences Finder (SSF, http://www.umd.be/searchSpliceSite.html (accessed on 18 February 2021)), [66] SpliceAI (https://github.com/Illumina/SpliceAI (accessed on 18 February 2021)), [67] Sorting Intolerant From Tolerant (SIFT: values ≤ 0.05 are predicted pathogenic), [68] Polymorphism Phenotyping v2 HumVar (PolyPhen-2: values ≥ 0.09 are predicted pathogenic); [69] Align GVGD combines the biophysical characteristics of amino acids and protein multiple sequence alignments to predict where missense substitutions in genes of interest fall in a spectrum from enriched deleterious to enriched neutral (Class C65 is the most likely to interfere with protein function), [70] Mutation Taster (MT, values ≥ 0.5 are predicted pathogenic), [71] Revel (predicts pathogenicity of missense variants on the base of multiple individual tools).
